# The relationship between family communication and family resilience in Chinese parents of depressed adolescents: a serial multiple mediation of social support and psychological resilience

**DOI:** 10.1186/s40359-023-01514-7

**Published:** 2024-01-18

**Authors:** Yinying Zhang, Yiwen Hu, Min Yang

**Affiliations:** Xiangya Nursing School of Central South University, Changsha, Hunan Province People’s Republic of China

**Keywords:** Major depressive disorder, Adolescent, Family communication, Family resilience, Social support, Psychological resilience

## Abstract

**Background:**

Family resilience plays a crucial role in helping depressed adolescents overcome challenges. However, studies examining family resilience in depressed adolescents are currently scarce. This study, guided by the family resilience framework, aimed to investigate the serial-multiple mediation of social support and psychological resilience between family communication and family resilience in Chinese families of depressed adolescents.

**Methods:**

In 229 parents of adolescents with major depressive disorder, 20.1% comprises of fathers, while 79.9% comprises of mothers. The mean age of depressed adolescents was 14.84 (±1.76) years, and the mean age of parents of these depressed adolescents was 43.24 (±4.67) years. The Family Resilience Assessment Scale (FRAS), the Psychological Resilience of Parents of Special Children Questionnaire, and the Social Support Rating Scale, Family Assessment Device (FAD) were used to collected data. Descriptive, univariate, and Pearson correlation analyses were used in preliminary analyses. To explore mediation, we employed a serial-multiple mediation model (PROCESS model 6).

**Results:**

Family communication was positively correlated with family resilience, social support, and psychological resilience. Mediation analysis revealed indirect effects of family communication on family resilience, which were mediated solely by either social support or psychological resilience, or through multiple mediation pathways involving both social support and psychological resilience.

**Conclusions:**

Family communication positively and directly affects the family resilience of depressed adolescents, and a higher level of social support and psychological resilience can help improve family resilience. These findings not only provide empirical evidence supporting the family resilience framework but also have practical implications for future family interventions targeting depressed adolescents.

## Introduction

Depression is one of the most common mental health problems among adolescents. Over the past three decades, the prevalence of adolescent depression dramatically increased in China [1]. A recent large-scale nationwide epidemiological survey in China found a 3.0% prevalence of depression among children and adolescents [2]. Adolescent depression is a chronic illness with a recurrent relapse process and a high risk of suicide [3, 4], many adolescents are compelled to discontinue their education as a result. Relevant surveys have indicated that 41% of adolescents drop out of school and stay at home after suffering from depression [5], leading to their isolation from society. In turn, parents, as the primary caregivers of adolescents, face immense challenges in guiding the socialization of adolescents. A large body of prior research has investigated the negative impact of adolescent depression on families. Long-term hospitalization places a heavy financial burden on families, especially for some low-income families [6]. In addition, families face long-term caregiving burdens, family dysfunction, and breakdown of family relationships, severely reducing the quality of life of family members [7–9]. Notably, a recent study found that some families with chronically ill children demonstrate satisfactory coping skills, and use their experiences to facilitate normal family functioning [10].

Walsh proposed that all families have the potential for growth and repair in the face of misfortune, threat, trauma or crisis [11], which was the concept of family resilience. Promoting family resilience in families with chronically ill children can yield many positive outcomes. Studies have found that family resilience is strongly associated with the flourishing growth of children in adversity [12]. Although previous studies have indicated that family resilience program can effectively reduce post-traumatic stress and negative emotions in parents, while improving overall family functioning [13], studies examining the mechanism of family resilience in depressed adolescents are currently inadequate [9, 14]. There are some researches to explore the mechanism of resilience, but they are focusing on individual resilience rather than family resilience [15, 16].

This research is based on the Family Resilience Framework [17, 18]. The framework emphasizes that family resilience is a dynamic process with three main processes: communication progresses, organizational patterns, and belief systems [17]. Communication progresses are the processes of clarifying ambiguous information and seeking consistency between words and actions within the family, such as family communication. Organizational patterns are the use of social resources to gain mutual support and connectedness among family members, such as social support. Belief systems are the attitudes of families in the face of unexpected events and adversity, such as psychological resilience of family members. Walsh argued that these three factors are mutually interactive, and sustain each other over time to promote family resilience [17]. However, few studies have explored the potential mechanisms by which these three factors enhance family resilience through their interactions [19].

Prior researches have demonstrated that family communication is an important predictor in promoting family resilience [20, 21]. Good communication is defined as the clear transmission of information, open emotional sharing, and the collaborative resolution of difficulties by Walsh [17]. The definition of good communication might be different in different culture, but the results of a research have demonstrated that the young generation in China are undergoing a dynamic shift from high-context to low context culture due to the globalization [22]. Good communication between parents of depressed adolescents is beneficial in stabilizing family functioning when faced with shared parenting stress [7]. Nevertheless, there is a scarcity of studies examining the impact of parent-child communication on family resilience [23]. Family communication is undoubtedly a great challenge for parents and children who is in adolescent rebellion [24, 25]. Furthermore, family communication not only can directly influence family resilience, but also can impact family resilience by mediators, as outlined in the family resilience framework [26]. However, there is still a lack of studies that have provided a detailed explanation of this mechanism.

Some studies have suggested that social support plays a crucial role in promoting family resilience [27, 28]. A recent meta-analysis found a direct link between social support and the mental health of parents with adolescents suffering from depression [29], indicating that social support may have a positive impact on these family. A qualitative study examining families of children with sickle cell disease found that effective family communication enhances access to both internal and external family resources during times of adversity, ultimately bolstering family resilience [30]. Based on these analyses, social support could potentially serve as a mediating variable between family communication and family resilience in depressed adolescents. However, the literature exploring the mediating role of social support in the relationship between family communication and family resilience among depressed adolescents remains limited.

Another key protective factor of family resilience is psychological resilience. Psychological resilience refers to the process by which individuals adapt well in the face of adversity, trauma, tragedy, or significant threats [31]. Promoting psychological resilience in parents of children with chronic illness is one aspect of psychosocial care that can affect the well-being of the entire family [32]. Previous empirical research has generally considered family resilience to be an influencing factor in psychological resilience rather than an outcome variable [33, 34]. However, Walsh’s theory explicitly identifies psychological resilience as an important contributor to family resilience [17, 18], few empirical studies have confirmed this hypothesis.

Theiss [35] suggested that good family communication helps increase psychological resilience of parents and their children. Therefore, psychological resilience is not only directly associated with family resilience but also holds the potential to serve as a mediation between family communication and family resilience. In addition to family communication, social support has been shown to be vital in promoting mechanisms of psychological resilience among parents who have lost an only child [36]. According to the Kumpfer’s resilience theory framework, challenges activate the resilience process, and then the promotion of the internal factor (psychological resilience) through the external environmental factor (social support) can facilitate the development of resilience [37]. High levels of social support can enhance individuals’ sense of belonging and self-confidence, prompting them to adopt more positive ways to cope with stress, and enhancing their capacity to regulate negative emotions such as anxiety and depression [38, 39]. The available evidence raises the possibility that social support and psychological resilience may serve as serial mediators in the relationship between family communication and family resilience among Chinese parents of depressed adolescents. However, the literature directly supporting this inference remains inadequate.

This study aimed to explore the serial-multiple mediation of social support and psychological resilience in the relationship between family communication and family resilience based on the Family Resilience Framework [17, 18]. Based on the above literature, we proposed three hypotheses: 1) family communication is positively associated with family resilience. 2) family communication indirectly predict family resilience through the independent mediating effect of social support and psychological resilience. 3) social support and psychological resilience are two serial mediators in the relationship between family communication and family resilience.

## Methods

### Design and participants

This cross-sectional study was a part of a larger mixed methods project. This project quantitatively investigated the relationships between family resilience and family communication in parents of depressed adolescents in this paper, and in another paper, interpretative phenomenological analysis method was used to explore the dynamic processes of family resilience from parents of adolescents with depression [9]. Participants were recruited from the inpatient department of children and adolescent psychiatry of two tertiary hospitals in Changsha, China, between May and December 2020. Convenience sampling was used to recruit participants who met the following criteria: (1) biological parents of an adolescent (12–18 years old) diagnosed with major depressive disorder (International Classification of Disease, Tenth Version F32.2–32.3); (2) voluntary participation in this study; (3) able to comprehend and complete the questionnaires relevant to this study. The Ethical Committee of the Nursing School, Central South University, approved this research (protocol code: E202005; date of approval: 18 March 2020) in accordance with the ethical principles of the Helsinki Declaration. Prior to participating in the survey, all participants were informed of the purpose and content of the study, and they participated voluntarily and were able to withdraw from the study at any time.

The desired sample size was calculated to be 134 using the G*Power 3.1 program, with a medium effect size of 0.30, a significance level of 0.05, and power of 0.95 [40]. Considering a 20% missing rate, a sample size of 168 was required for this study. Of the 285 eligible participants who agreed to participate in this study, 229 completed the questionnaires, representing an 80.3% response rate. Fifty-six samples were excluded due to incomplete questionnaires. There was no significant difference in general information between the 56 excluded samples and the 229 samples.

### Data collection

Data were collected using a structured questionnaire administered by a PhD student with experience studying research related to adolescent depression. Prior to the study, all participants were informed of the purpose and procedures of the study and were informed that the results would be reported anonymously. After signing the informed consent form, participants were asked to complete a series of questionnaires that took 15–20 minutes to complete. Throughout the process of completing the questionnaire, the researchers were in the same room as the participants, providing explanations and assistance. During data collection, the research team verified the completeness of the questionnaire information daily and entered the data into Epidata version 3.1.

### Measures

The general information questionnaire includes socio-demographic characteristics relevant to family resilience, formulated through a review of literature and discussions within our research group. These data are collected to explore the differences in the distribution of socio-demographic characteristics within the sample of this study, and to mitigate the influence of confounding variables in subsequent mediation analysis due to the convenience sampling employed in this study. General characteristics of adolescents with depression encompassed gender, age, duration of diagnosis, first-episode, suicide history, family types, and sibling health status. Correspondingly, relevant characteristics of parents comprised of relationship with patients, age, education, employment, and monthly family income.

The Family Resilience Assessment Scale (FRAS) was used to assess the resilience levels of parents of adolescents with depression [41]. The Chinese version of the FRAS, which was adapted by Dong Chaoqun et al., was employed [42]. The FRAS includes 44 items across four domains: family communication and problem-solving (e.g., “we are understood by other members of the family”), utilization of socioeconomic resources (e.g., “we are aware that help from the community is always available in times of difficulty”), maintenance of positive attitudes (e.g., “in the face of significant challenges, we feel empowered”), and attribution of meaning to adversity (e.g., “we accept stressful events as a part of life”). Participants completed the Chinese version of the FRAS (C-FRAS) using a Likert 4-point scale, 1 = “strongly disagree”, 2 = “disagree”, 3 = “agree”, 4 = “strongly agree”, with total scores ranging from 44 to 176, and higher scores indicating greater family resilience. The total C-FRAS scale showed strong internal consistency (Cronbach’s alpha coefficient = 0.96), as did the subscales (Cronbach’s alpha coefficients ranged from 0.70 to 0.97).

The psychological resilience of parents of adolescents with depression was assessed using the Psychological Resilience of Parents of Special Children Questionnaire [43], which consists of 26 items across six dimensions: problem-solving skills (6 items), spiritual support (5 items), sense of control (5 items), optimistic resilience (4 items), self-confidence (3 items), and acceptance (3 items). Responses were rated on a 5-point Likert scale ranging from “very unlikely” to “very likely”, with higher scores indicating greater psychological resilience. The Cronbach’s alpha coefficient of the total questionnaire in this study was 0.887.

The Social Support Rating Scale, developed by Xiao Shuiyuan in 1994 [44], assesses the social support of parents with depressed adolescents using three dimensions, including objective support (for example, “What are the sources of solace and care you receive when encountering emergency situations?”), subjective support (for example, “How many intimate friends do you have who can offer support and assistance?”), and the utilization of support (for example, “What are the methods you employ to seek assistance when encountering troubles?”). For items 1–4 and 8–10, respondents select only one option, with scores ranging from 1 to 4. For item 5, scores range from 1 to 4, with each item ranging from “none”, “very little”, “average”, and “low”, and are based on five sub-items. For items 6 and 7, a response of “no sources” is scored as 0, if the response is “the following sources exist,” then the number of sources is counted as the score. The total score ranges from 12 to 66, with scores < 22, 23–44, and 45–66 indicating respectively low, medium, and high levels of social support. The Social Support Rating Scale has demonstrated good reliability and validity among the Chinese population [45]. The scale has high internal consistency with a Cronbach’s alpha coefficient of 0.92.

The Family Communication Scale, a subscale of the Family Assessment Device (FAD), was utilized to evaluate parental communication within families with depressed adolescents [46]. The questionnaire comprises 9 items, for example, the thoughts of my family members are never disclosed to other family members. Responses are rated on a 4-point Likert scale ranging from “strongly agree” to “completely disagree”, with total scores ranging from 9 to 36 points, lower scores indicate better family communication. The Cronbach’s alpha coefficient of the scale in this study was 0.72.

### Statistical analyses

The IBM SPSS software version 25.0 was utilized to analyze the data. Descriptive statistics, independent t-tests, one-way ANOVA, and Kruskal–Wallis H test were performed to describe and compare the family resilience among Chinese parents of adolescents with depression based on the sociodemographic characteristics. If the data analysis results indicate that the distribution of certain sociodemographic characteristics variables differs significantly within our study sample (P<0.05), these variables will be included as covariates in subsequent chain mediation analysis to mitigate the confounding effects on the research outcomes. Pearson correlation analysis was conducted to assess the relationship between family communication, social support, psychological resilience, and family resilience. The three hypotheses (H1, H2, H3) were tested using a serial mediation model (model 6) of the PROCESS macro v4.2 in SPSS. This approach employed an ordinary least-square regression model and the bootstrap method to estimate of the indirect effect and of its 95% confidence intervals (CIs) through random and resampling techniques, which better controlled on type I errors. In this study, 5000 bootstrap samples were employed to examine the indirect effect of one independent variable (family communication), two mediators (social support and psychological resilience), and one dependent variable (family resilience). Furthermore, the general characteristics related to family resilience were tested as covariates. A significance level of .05 (two-tailed) was set.

Potential common method bias may exist as the same person completed the survey questionnaires in this study. To minimize the impact of common method bias, we implemented standardization procedures. These measures included setting appropriate length of the questionnaire, ensuring anonymity of respondents, and implementing reverse scoring for certain scale items. To assess the common method bias, Harman’s single-factor test was employed, as has been commonly done in previous research [47, 48]. Harman’s single-factor test involves conducting an exploratory factor analysis with all variables included and identifying the presence of significant common method bias if only one factor is extracted or if a certain factor has particularly strong explanatory power. The analysis results indicate that the first factor accounts for 22.86% of the variance, which is significantly lower than the critical value of 40%. Therefore, the potential issue of common method bias is not considered significant in this study.

## Results

### Preliminary analyses

In the current sample (*N* = 229), the mean age of depressed adolescents was 14.84 (±1.76) years (range 12–18). The majority of depressed adolescents were female (74.2%, *N* = 170), experienced depression for more than one episode (57.6%, *N* = 132), had a duration of less than 12 months (44.5%, *N* = 102), had a suicide history (59.0%, *n* = 135), and had healthy siblings (62.9%, *N* = 144). Additionally, the mean age of parents of the 229 depressed adolescents was 43.24 (±4.67) years (range 31–55), with mostly mothers (79.9%, *N* = 183), educational attainment of the middle school or below (40.6%, *N* = 93), and employment status (56.8%, *N* = 130). Further characteristics of the participants can be found in Table 1.

**Table 1:** Differences in family resilience by general characteristics (*N* = 229)

Characteristics	Family resilience (M ± SD)	N (%)	t/F	*p*-value
General characteristics of adolescents with depression				
Gender			0.342	0.732
Male	132.69 ± 18.60	59 (25.8)		
Female	131.72 ± 18.82	170 (74.2)		
Age (years)			−1.014	0.311
12–15	131.05 ± 18.33	149 (65.1)		
16–18	133.68 ± 19.46	80 (34.9)		
Time since diagnosis (months)			1.195	0.304
≤ 12	130.38 ± 18.11	102 (44.5)		
13–24	131.50 ± 17.70	62 (27.1)		
≥ 25	134.92 ± 20.50	65 (28.4)		
First-episode ^a^			0.324	0.746
Yes	132.44 ± 17.42	97 (42.4)		
No	131.62 ± 19.69	132 (57.6)		
Suicide history ^b^			0.319	0.750
Yes	132.30 ± 18.66	135 (59.0)		
No	131.50 ± 18.92	94 (41.0)		
Family types			3.944	0.021
Original family	133.33 ± 18.32	189 (82.5)		
Stepparent family	130.41 ± 17.00	17(7.4)		
Single-parent family	121.95 ± 20.87	23(10.0)		
Sibling’ s health status			3.545	0.030
Only child	133.20 ± 20.42	77 (33.6)		
Having healthy siblings	132.25 ± 17.73	144 (62.9)		
Having unhealthy siblings	115.00 ± 11.32	8 (3.5)		
General characteristics of parents				
Relationship			2.507	0.014
Father	137.82 ± 17.42	46 (20.1)		
Mother	130.50 ± 18.80	183 (79.9)		
Age (years)			0.258	0.772
≤ 40	130.68 ± 19.51	74 (32.3)		
41–50	132.54 ± 18.39	139 (60.7)		
≥ 51	132.93 ± 18.82	16 (7.0)		
Education			2.450	0.089
Middle school and below	129.03 ± 19.24	93 (40.6)		
High school	132.37 ± 17.89	67 (29.3)		
Bachelor or above	135.55 ± 18.43	69 (30.1)		
Employment			2.274	0.024
Employed	134.40 ± 18.57	130 (56.8)		
Unemployed	128.77 ± 18.55	99 (43.2)		
Monthly family income (yuan)^c^			1.415	0.239
≤ 3000	131.41 ± 23.66	39 (17.0)		
3001–5000	130.55 ± 18.60	78 (34.1)		
5001–8000	130.11 ± 14.82	59 (25.8)		
≥ 8001	136.54 ± 18.52	53 (23.1)		

^a^ First diagnosed with depression by a psychiatrist

^b^ the history of non-suicidal self-injurious behavior or suicide attempts

^c^ 3000 Chinese Yuan ≈ 459.77 US dollar; and the national per capita disposable annual income of Chinese residents in 2020 was 32,189 Chinese Yuan (http://www.gov.cn/guoqing/2021-04/09/content_5598662.htm)

In this study, the mean score for family resilience was 131.97 ± 18.73. The mean score for psychological resilience was 101.31 ± 12.71, problem solving skills received the highest rating, while self-confidence received the lowest score. The mean score for social support was 39.70 ± 7.42, and the mean score for family communication was 20.37 ± 3.42. Independent samples t-test revealed significant differences in family resilience between parents of depressed adolescents by their relationship with the patient (t = 2.422, *p* = 0.016) and employment status (t = 2.307, *p* = 0.022). Furthermore, one-way analysis of variance demonstrated significant differences in family resilience among parents of depressed adolescents by family type (F = 3.784, *p* = 0.024). Kruskal-Wallis H test indicated significant differences in family resilience among parents of depressed adolescents by their sibling’ s health status (*p* = 0.017) (see Table 1). If the data analysis results indicate that the distribution of certain sociodemographic characteristics variables differs significantly within our study sample (P<0.05), these variables will be included as covariates in subsequent chain mediation analysis to mitigate the confounding effects on the research outcomes.

### Preliminary correlation analyses

Pearson’s correlation analysis revealed that family communication exhibited a significant negative correlation with family resilience (r = − 0.560, *p* < 0.001), social support (r = − 0.246, *p* < 0.001), and psychological resilience (r = − 0.421, *p* < 0.001). Since the Family Communication Scale is reverse scored, with lower scores indicating higher levels of family communication, improvement in family communication will enhance the levels of social support and psychological resilience. Family resilience exhibited a positive correlation with social support (r = 0.400, *p* < 0.001) and psychological resilience (r = 0.590, *p* < 0.001). Furthermore, social support showed positive correlation with psychological resilience (r = 0.397, *p* < 0.001) (shown in Table 2).

**Table 2:** Pearson’s correlations test between family communication, family resilience, social support and psychological resilience (*N* = 229)

Variables	Mean (SD)	1	2	3	4
1 Family communication	20.37 ± 3.42	1.000			
2 Family resilience	131.97 ± 18.73	−0.560	1.000		
3 Social support	39.70 ± 7.42	−0.246	0.400	1.000	
4 Psychological resilience	101.31 ± 12.71	− 0.421	0.590	0.397	1.000

All values statistically significant at *p* < .01 (two-tailed). The Family Communication Scale is scored in reverse, where lower scores indicate better family communication. The Family Communication Scale is scored in reverse, with lower scores indicating higher levels of family communication

### Mediation analyses

**Fig. 1:**
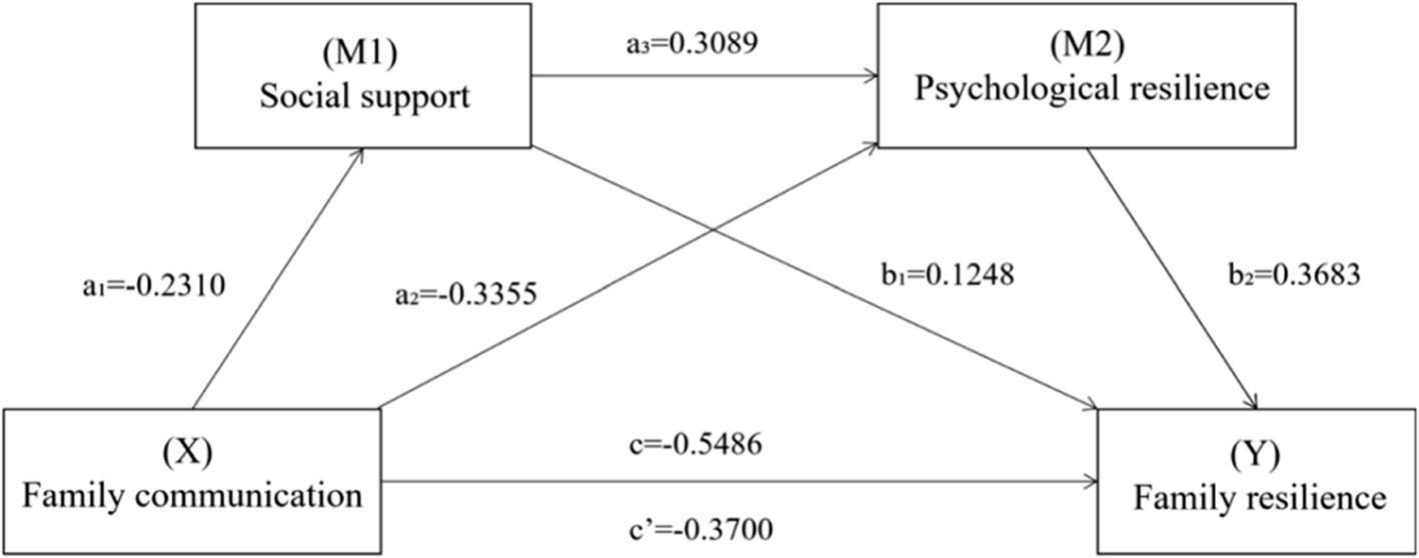
Hypothesized serial mediation model linking family communication and family resilience through social support and psychological resilience as serial mediators

As shown in Fig. 1, the serial multiple mediation model indicated that family communication, social support, and psychological resilience explained 50.7% of the variance in family resilience (R^2^ = 0.507, F = 32.43, *p* < 0.001). The total effect (c = − 0.55, SE = 0.29, t = − 10.27, *p* < 0.001) of family communication was found to have a significant positive effect on family resilience. In addition, family communication was positively associated with social support (a_1_ = − 0.23, SE = 0.13, t = − 3.83, *p* < 0.001) and psychological resilience (a_2_ = − 0.34, SE = 0.21, t = − 5.84, *p* < 0.001). Social support was positively associated with psychological resilience (a_3_ = 0.31, SE = 0.11, t = 4.99, *p* < 0.001). The direct effects of social support (b_1_ = 0.12, SE =0.14, t = 2.24, *p* = 0.003) and psychological resilience (b_2_ = 0.37, SE = 0.08, t = 6.44, *p <* 0.001) on family resilience were significant. When family communication and the two mediating variables (social support and psychological resilience) were included in the model simultaneously, the direct effect of family communication on family resilience remained significant (c′ = − 0.37, SE = 0.29, t = − 7.03, *p* < 0.001). In summary, these results support the occurrence of a serial multiple mediation model.

As seen in Table 3, after adjusting for all covariates, including family types, employment, the relationship with patient, and sibling’ s health status, the path through single mediation of social support (point estimate = − 0.03, 95% CI -0.06, − 0.001), the path through single mediation of psychological resilience (point estimate = − 0.12, 95% CI -0.18, − 0.08), and the path through both mediators (point estimate = − 0.03, 95% CI -0.05, − 0.01) were all statistically significant. The total indirect effect also showed statistically significant (point estimate = − 0.18, 95% CI -0.24, − 0.12). As a result, the path through both mediators was significant; and the indirect effect through both social support alone and psychological resilience alone was also significant.

**Table 3:** Bootstrapping indirect effects and 95% confidence intervals for the final mediational model (*N* = 229)

	Point estimate	Boot SE	Boot LLCI	Boot ULCI
Effect				
Total indirect effect of X on Y	−0.1786	0.0311	−0.2437	− 0.1199
Indirect effect 1: X → M1 → Y	−0.0288	0.0157	−0.0624	− 0.0012
Indirect effect 2: X → M2 → Y	−0.1236	0.0273	−0.1811	− 0.0752
Indirect effect 3: X → M1 → M2 → Y	−0.0263	0.0097	−0.0481	− 0.0108
Contrasts				
Model 1 versus Model 2	0.0948	0.0350	0.0267	0.1658
Model 1 versus Model 3	−0.0025	0.0184	−0.0380	0.0363
Model 2 versus Model 3	−0.0973	0.0271	−0.1543	−0.0478

*X *family communication, *M1 *social support, *M2 *psychological resilience, *Y *family resilience. *Model 1 *family communication→ social support→ family resilience, *Model 2 *family communication→ psychological resilience→ family resilience, *Model 3 *family communication→ social support→ psychological resilience →family resilience, *SE *standard error, *CI *confidence interval, *LL *lower level, *UL *upper level. The Family Communication Scale is scored in reverse, with lower scores indicating higher levels of family communication

To evaluate the relative strength of indirect effects of mediators, we examined three paths (X → M1 → Y; X → M2 → Y; X → M1 → M2 → Y). The path through single mediation by social support (X → M1 → Y) had a stronger mediating effect than the path through single mediation by psychological resilience (X → M2 → Y). The path through the serial-multiple mediation (X → M1 → M2 → Y) had a stronger mediating effect than the path through single mediation by social support (X → M1 → Y). Moreover, there was no significant difference between the path through single mediation by social support (X → M1 → Y) and the path through the serial-multiple mediation (X → M1 → M2 → Y).

## Discussion

In this study, the average family resilience score of 229 parents of adolescent depression patients was 131.97 ± 18.73 (total score ranging from 44 to 176), with item scores averaging 2.99 ± 0.42 (out of a total of 4). This result indicates that their family resilience is at a moderately high level. The average score for family communication was 20.37 ± 3.42, with average item score 2.26 ± 0.38. According to the McMaster critical value judgment method [49], if the average score for family communication items exceeds 2.2, it indicates that the family has communication issues, parents of depressed adolescents in our study indeed have issues with poor family communication. The results also showed that 70.8% of parents of depressed adolescents are at a moderate level of social support, with average score 39.70 ± 7.42. The average score for psychological resilience was 101.31 ± 12.71 (ranging from 26 to 130) with the average item score 3.89 ± 0.48 (out of a total of 5), which indicates that their psychological resilience is at a moderately high level.

This study also investigated the association between family communication and family resilience among depressed adolescents in China, while examining the mediating roles of social support and psychological resilience. The findings indicated that improving family communication can enhance family resilience directly and indirectly, through social support and psychological resilience, as well as through the multiple mediating effects of social support on psychological resilience.

In line with prior studies [21, 50, 51], family resilience was also influenced by family communication directly in families of adolescents with depression. Children with depression often experience negative emotions and may feel nervous and irritable during family communication. This can hinder parents from understanding the difficulties and stresses of their children, and may impede family resilience and adjustment during hardships [52]. Conversely, active and effective family communication, such as active listening, providing positive feedback, and considering others’ perspectives, can facilitate problem-solving and help family members to overcome challenges together [53]. On the other hand, families with strong communication skills tend to exhibit higher levels of cohesion and adaptability [54], which in turn can help maintain family harmony and stability even in the face of adversity.

The results of this study supported the hypothesis that social support was a mediator of the association between family communication and family resilience. Family members are the closest and most trusted people for adolescents with depression [55]. Families with good communication always exhibit trust and satisfaction with each other, which facilitates better understanding and support among family members [56]. Specifically, effective family communication can help adolescents with depression gain more social support and alleviate their depressive symptoms, such as guiding them to develop good social skills, building supportive relationships with friends, and seeking professional psychological counseling when necessary [57, 58]. Furthermore, promoting social support, including good family relationships, intimacy with parents, peer support, and teacher support, can help alleviate family stress [59, 60], which may prove to be a promising goal for enhancing family resilience for adolescents with depression.

The findings of this study also supported the hypothesis that psychological resilience is another important mediator between family communication and family resilience. Previous research has shown that parents of depressed adolescents are more likely to suffer from depression and poorer mental health when compared to control groups [61]. Moreover, they are less able to maintain positive emotions when faced with adversity or challenges. Family communication patterns theory suggests that family members with a high propensity for dialogue have better emotional well-being and are more adaptable and competent in dealing with challenges than families with other communication types [62], which also implies a higher level of psychological resilience. Additionally, according to Walsh’s family resilience framework, the psychological resilience of family members can act as a promotive factor for family resilience [17]. Families of depressed adolescents face numerous challenges such as their adolescent’s emotional problems, difficulties with family relationships, and academic stress [63]. Family members with higher psychological resilience are better equipped to overcome these challenges and maintain their own and their family’s health [64]. This contributes to the overall family resilience, enabling the family to cope with the stresses of life.

The findings also confirm that the relationship between family communication and family resilience is mediated by the effect of social support on psychological resilience. Firstly, effective family communication positively influences the availability of social support for families with depressed adolescents [57]. In addition, numerous studies have found a positive association between social support and psychological resilience [38, 65–67]. Some studies have suggested that individuals with higher levels of social support tend to have a greater sense of control and self-confidence, which can enhance their resilience in the face of stress, and promote the adoption of positive coping strategies to reduce stress and increase psychological resilience [68, 69]. Finally, family members with high levels of psychological resilience are more capable of addressing the challenges in life effectively, which in turn promotes greater family resilience [64].

### Clinical implications

The results of this study confirm that the level of family resilience is significantly lower among parents of adolescents with depression than parents of children with other chronic illnesses [70]. Family resilience is strongly associated with promoting health of child and mitigating the negative effects of adversity [12]. Therefore, it is crucial to clarify the factors that foster family resilience and to explore interventions aimed at enhancing family resilience in depressed adolescents. The main finding of this study highlights that family communication not only have a direct influence on the family resilience of depressed adolescents, but also have an indirect influence on family resilience through social support and psychological resilience. Psychiatric nurses, who provide round-the-clock care for depressed adolescents in hospital, play a critical role in promoting family resilience. They can invite parents with strained family relationships to attend lectures on family communication skills, aiming to enhance the bond among family members. Additionally, psychological support can be established within the ward to offer professional guidance to parents dealing with emotional burdens, enhancing their psychological resilience and enabling them to actively participate in the care of their adolescents.

Creating a supportive environment is also essential for promoting family resilience. Psychiatric doctors and nurses can provide social support by offering professional knowledge about mental health, teaching emotional management skills, and providing emotional support. In addition, this study identified several potential factors that may influence the resilience of families with depressed adolescents, including family types, the health status of siblings, the relationship with adolescents, and the education and employment levels of parents. Psychiatric doctors and nurses can also perform psychological and social assessments of the patients’ families and provide guidance to those who may need further support, by referring them to social support groups or charitable organizations that can offer assistance.

### Limitations

Firstly, this study is a cross-sectional study, which impedes us to establish the causal relationship among the four variables. Additionally, the study design also restricts us to investigate family resilience over time. To overcome this limitation, future research on this topic should consider conducting longitudinal studies. Secondly, our study was conducted in the psychology departments of two hospitals in China, and a majority of our sample consisted of females. Therefore, the generalizability of our findings may be limited by the representativeness of our sample. Thirdly, this study utilizes the framework of family resilience to address adolescent depression, yet family resilience may also play a significant role in the development of depression. Future research could consider exploring the protective effects of family resilience on adolescents with a history of adverse childhood experiences and depression. Fourthly, in this study all questionnaires were answered by parents, which may not fully accurately reflect the true situation of family resilience in depressed adolescents. To address these issues, future research could simultaneously collect the data from both adolescents and parents, in order to obtain more comprehensive results from different perspectives. Finally, this study has a significant limitation that convenience sampling was utilized for data collection. Despite the inclusion of confounding variables in the data analysis to alleviate the impact of convenience sampling on the research findings, such influence cannot be entirely eliminated. Future research could employ a more rigorous random sampling approach and consider increasing the inclusion of families from different regions as samples, thus enhancing the reliability of the results.

## Conclusion

Firstly, this study confirms that family communication, social support and psychological resilience can significantly and positively predict family resilience among family with depressed adolescents. Secondly, family communication can not only directly predict family resilience, but also indirectly predict family resilience through the independent mediating effect of social support and psychological resilience, and indirectly predict family resilience through the chain mediating effect of social support and psychological resilience. This study explores the mechanisms of family resilience, enriching the content of the Family Resilience Framework, and provides new perspectives for future family resilience intervention programs for parents of adolescents with depression.

## Data Availability

Data available on request due to restrictions eg privacy or ethical.
